# Pulmonary granulomas in a patient with positive ANCA and history of tuberculosis: case report

**DOI:** 10.1186/s12890-020-01258-9

**Published:** 2020-08-14

**Authors:** B. Wong, E. Tan, A. McLean-Tooke

**Affiliations:** 1 Dept of Respiratory Medicine, Sir Charles Gairdner Hospital, Nedlands, 6009 Australia; 2 Department of Clinical Immunology, Sir Charles Gairdner Hospital, Nedlands, 6009 Australia

**Keywords:** Tuberculosis, PR3-ANCA, Granulomatous Polyangiitis, Case report

## Abstract

**Background:**

Granulomatous polyangiitis (GPA) is a rare multisystem autoimmune disease of unknown aetiology that is pathologically characterised by necrotising vasculitis, tissue necrosis and granulomatous inflammation, typically in the presence of anti-neutrophil cytoplasmic antibodies (ANCA). However infectious diseases may induce high titre ANCA and mimic vasculitis. Tuberculosis may share many clinical features with GPA including fever, arthralgia, granulomatous inflammation and pulmonary lesions and patients.

**Case presentation:**

A 39 year old patient was admitted with ocular irritation and redness, arthralgia and multiple new pulmonary lesions. The past medical history was significant for two episodes of tuberculosis previously requiring prolonged treatment. ANCA antibodies were positive and CT showed multiple pulmonary lesions including cavitatory lesions. After extensive investigation, the patient was treated for GPA with high dose immune suppression with good clinical response.

**Conclusions:**

Here we review the diagnostic considerations between differentiating GPA and tuberculosis in patients from endemic regions. It is recommended that biopsies of lung lesions, sputum microscopy and multidisciplinary team input are sought as part of the workup when these two differentials are being considered.

## Background

Granulomatosis with polyangiitis (GPA), formerly known as Wegener’s granulomatosis, is a form of systemic vasculitis of unknown aetiology that predominantly involves small vessels and can affect the arteries, veins or capillaries [1–3]. GPA is rare, with a reported incidence of up to 11.8 per million person-years, with an increased frequency described in Caucasians or those of European ancestry [2, 4, 5]. GPA typically involves the upper respiratory tract, lower respiratory tract and kidney, however virtually any organ can be affected including the skin, eyes, and central or peripheral nervous system [6, 7]. Pulmonary features may include multiple bilateral cavitating lesions, nodules, infiltrates or diffuse alveolar haemorrhage, whilst otolaryngologic features include nasal septal perforation, hearing loss and subglottic stenosis [2, 6]. Renal involvement can present as a rapidly progressive glomerulonephritis (RPGN) with acute renal impairment and haemoproteinuria [8]. Histologically, GPA is characterised by necrotising granulomatous inflammation and necrotising vasculitis involving the small vessels [1]. Serologically, GPA is associated with the presence of anti-neutrophil cytoplasmic antibodies (ANCA) [2]. Diagnosis can be challenging, as the presentation of GPA is heterogenous in terms of severity, number of organs involved, and tempo of disease. Prompt treatment with aggressive immunosuppression is required to prevent long-term sequelae such as irreversible renal injury, severe large airway stenosis and visual loss [7]. Therapy is able to induce remission in up to 90% of patients with GPA, however unfortunately 38% of patients relapse within 5 years [9].

ANCAs are considered highly specific for the ANCA-associated vasculitides (AAV) which include GPA, eosinophilic granulomatosis with polyangiitis (EGPA; formerly known as Churg-Strauss syndrome) and microscopic polyangiitis (MPA) [3, 8]. ANCAs are antibodies directed against proteins located within cytoplasmic granules of neutrophils and monocytes [8, 10]. Two key ANCA patterns are described with indirect immunofluorescence (IIF) – cytoplasmic staining (C-ANCA), characterised by granular cytoplasmic staining; and peri-nuclear staining (P-ANCA), characterised by homogenous peri-nuclear staining [11]. Although autoantibodies against various different cytoplasmic proteins can produce these patterns, proteinase-3 (PR3) and myeloperoxidase (MPO) have been identified as the clinically relevant autoantigens most strongly associated with systemic vasculitides [8, 11–14]. In practice, ANCA are usually detected via initial screening with IIF using ethanol-fixed neutrophils, followed by testing of positive serum via enzyme-linked immunosorbent assay (ELISA) to confirm the presence of PR3-ANCA and MPO-ANCA. A positive C-ANCA or PR3-ANCA is reported in 80–90% of patients with active GPA and approximately 50% of patients with inactive disease; whilst a P-ANCA/MPO-ANCA has been reported in 3.6 to 15% of patients in cohorts with varied disease activity [10, 15, 16]. Conversely, MPO-ANCA is most frequently associated with EGPA and MPA [10]. Compared to GPA patients positive for MPO-ANCA, GPA patients positive for PR3-ANCA are more likely to experience clinical relapses and feature granulomatous lesions [10]. However, detection of ANCA are not required for diagnosis of AAV, and up to 20% of patients with active GPA are ANCA negative [15].

Multiple other conditions besides AAV have the capability to induce ANCAs. These include infections such as tuberculosis (TB), respiratory tract infections, endocarditis, malaria and leprosy; and medications such as propylthiouracil, minocycline, isoniazid and penicillamine [2, 3, 17–19]. TB and GPA exhibit overlapping pulmonary and extra-pulmonary features, including the presence of constitutional symptoms, cavitating lung lesions, arthralgias, and ocular manifestations such as uveitis [6, 20, 21]. Pulmonary necrotic granulomas in GPA can be difficult to distinguish radiologically from granulomas in the setting of pulmonary TB [17, 22, 23]. Therefore, TB infection-induced ANCA poses a particularly challenging diagnostic dilemma. Studies report the detection in ANCA up to 44% of patients with TB, with conflicting results in regards to the predominant ANCA pattern and confirmed specificities [17, 22, 23]. In addition, TB treatment itself may induce de-novo formation of ANCA, with studies demonstrating increased detection of ANCA post-TB treatment compared to pre-treatment [24, 25]. Given the overlapping clinical features but drastically different management strategies required for TB and GPA, a thorough and systematic clinical assessment along with multi-disciplinary radiological, histopathological, microbiological and serological results is required to obtain the correct diagnosis and initiate appropriate treatment.

We report a case of a patient who presented with multiple bilateral lung granulomas on a background of previously treated pulmonary TB, and describe the approach and investigations implemented to guide treatment.

## Case presentation

A 39-year-old female presented to a metropolitan tertiary hospital with a 1 week history of irritated and red eyes, with no associated eye pain or altered visual acuity. On further inquiry, 1 month prior to presentation she developed an episode of sinusitis and nasal crusting/erosions involving her nasal septum and left nostril. This was managed by her General Practitioner with a 5 day course of oral antibiotics (amoxicillin and clavulanic acid) and 7 days of 50 mg prednisolone daily. Her nasal erosions improved however her nasal congestion continued. She also had a 1 month history of fatigue and migratory arthralgias involving her wrist. There was no cough, dyspnoea, fever, night chills or weight loss.

Her past medical history was significant for previous TB infection. She had been treated in 1996 for pulmonary TB with a four drug regimen (2RHEZ/10RH), and in 2005 with a five drug regiment (2RHEZS/13HRZ) after being investigated for cervical lymphadenopathy. Subsequent to these treatments her TB was reported to be quiescent. Previous chest x-rays had showed bilateral apical calcified nodules.

The patient was born in India and moved to Australia in April 2017.

On examination she had bilateral conjunctival injection with normal visual acuity and extraocular eye movements. There were no rashes, no lymphadenopathy, no active synovitis, and no nasal lesions. General examination was unremarkable.

### Investigations

Blood tests showed elevated inflammatory markers with a C Reactive Protein of 72 (< 5 mg/L) and an Erythrocyte Sedimentation Rate of 104 (1-20 mm/hr). She had an eosinophilia of 1.6 (< 0.5X 10^9/L). PR3-ANCA was positive at 21 (< 2 U/ml).

Urine microscopy demonstrated an active urinary sediment with blood, protein and leucocytes detected. Albumin:creatinine ratio was elevated at 22.8 (< 3.5 mg/mmol) with a normal serum creatinine.

**Fig. 1:**
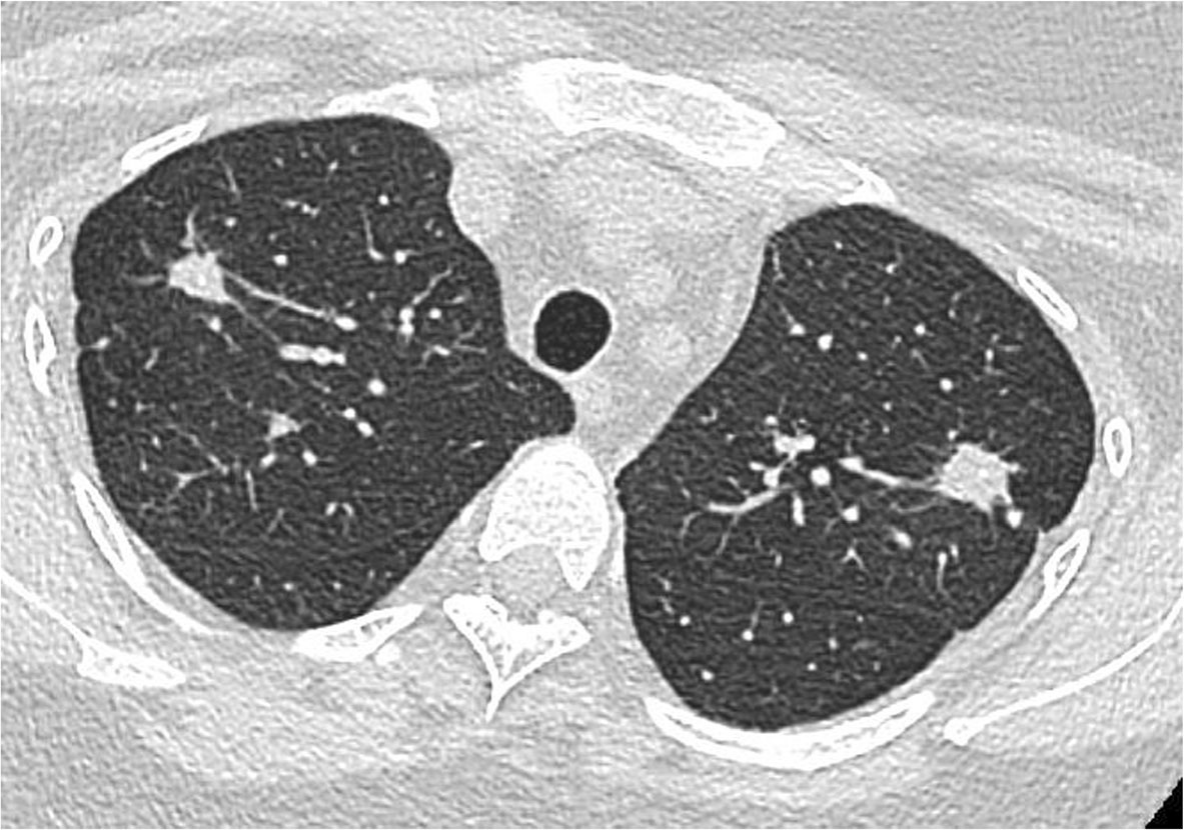
CT Chest of patient showing multiple granulomas

A chest X-Ray (CXR) confirmed new lesions within multiple lobes. A subsequent CT chest demonstrated multiple irregular calcified nodules in both lungs, with cavitation in a nodule in the right upper lobe (Fig. 1). A further nodule in the left upper lobe demonstrated adjacent cortical irregularity involving the rib. There was evidence of thoracic calcified lymphadenopathy.

Three separate samples of induced sputum were sent for microscopy, culture and sensitivities. This was negative with no acid fast bacilli seen. Bronchoscopy was considered but not performed due to the availability of CT guided lung biopsy at the centre. This enabled direct sampling of the lung lesions which would have a greater diagnostic yield over bronchoscopy. Numerous samples were obtained from multiple lesions to provide optimal tissue to investigate the differential diagnoses. This was done as a staged procedure by an experienced chest radiologist. From the left upper lobe lesion two fine needle aspirates and two core biopsies were performed. From the right middle lobe lesion two fine needle aspirates and three core biopsies were performed. Histopathological analysis demonstrated necrotising granulomatous inflammation with adjacent eosinophil-rich interstitial inflammation, organising pneumonia and sclerosis. No suppuration, infective organisms or definite vasculitis was identified. The fine needle aspirate samples were sent for cell block and microscopy, culture and sensitivities (MCS). No acid fast bacilli, fungi or pyogenic organisms were identified on special stains (Ziehl-Neelsen, Fite, PAS + D, Methenamine silver and Gram). Direct molecular testing by polymerase chain reaction (PCR) was positive for *Mycobacterium tuberculosis* complex DNA on the left upper lobe FNA sample.

From these investigations it was concluded that GPA was the most likely diagnosis given the negative sputum and core biopsy microscopy, clinical history of extended TB treatment and location of pulmonary nodules. The positive TB PCR was felt to be reflective of the previously treated TB rather than active infection.

### Treatment

The patient was induced with IV methylprednisolone (500 mg for 3 days) and two doses of rituximab (1 g two weeks apart). Cyclophosphamide was considered but decided against due to fertility concerns. She was then continued on oral prednisolone at 50 mg daily with a weaning regimen. On ophthalmological review her eye symptoms were attributed to a marginal keratitis and treated with fluorometholone eye drops.

### Outcome and follow-up

Following her initial induction treatment the patient had a good clinical response with improvement in her sinus and joint symptoms. CT chest at 3 months showed interval improvement with decrease in size multiple previous pulmonary masses and right upper lobe cavitation. Given a decreased Thiopurine methyltransferase (TPMT) level and thus mycophenolate was chosen over azathioprine as a steroid sparing agent. She had complete peripheral B cell depletion and normalisation of her PR3 within 4 months and her prednisolone dose was weaned down to 10 mg within 6 months of starting treatment. At 11 months after rituximab therapy she had B cell recovery associated with positive PR3-ANCA at 15 U/ml and clinical relapse with recurrence of ocular symptoms with uveitis and enlargement of pulmonary nodules on CT. Her corticosteroid dose was increased and she was retreated with 2 g of rituximab with resolution her ocular symptoms, normalisation of PR3-ANCA with interval improvement on CT and she remains under regular clinical review.

Her urine protein:creatinine ratio increased to 324 (< 13 mg/mmol) before stabilising at 60 mg/mmol with a normal renal function.

## Discussion and conclusions

This case illustrates the difficulty in distinguishing between tuberculosis and GPA given their similar clinical, radiological and histopathological features; with added complexity in this instance due to a confirmed previous history of pulmonary tuberculosis. Similarities between the two conditions highlighted by this case include the presence of cavitatory lung lesions, keratitis, granulomatous inflammation on biopsy and a positive PR3 ANCA. Thus to differentiate between the two conditions other diagnostic modalities needed to be considered including sputum analysis, lung biopsy and key aspects of the clinical history.

Features of this case supportive of a diagnosis of GPA over tuberculosis included her nasal symptoms and active urinary sediment which are not characteristic in tuberculosis. Aspects that perhaps favoured a diagnosis of tuberculosis included the fact that her pulmonary lesions were calcified and that one of them had associated rib erosion. One possible explanation would be that the patient’s GPA pulmonary lesions overlapped with her old tuberculosis lesions.

In regards to the positive TB DNA PCR that was identified, it has previously been described how false positives of a PCR assay can occur in the setting of nonviable mycobacterium [25], which would be in the case of previously treated disease. TB PCR has been demonstrated to be positive for many years after successful direct observed TB treatment [26, 27]. It is this inability for PCR to distinguish between viable and dead organisms that preclude TB PCR as a definitive test for active TB in the setting of prior TB infection.

The case draws to attention the overlap of autoantibodies in conditions such as tuberculosis and GPA. ANCA are considered highly specific for GPA, but the presence of TB infection-induced ANCA is a recognised phenomenon. Various studies have assessed the presence of circulating ANCA in patients with TB. Studies of patients with TB from Mexico, India, Iran and France reported detection of ANCA by IIF in 44, 30, 28 and 10% of patients respectively, with different predominant ANCA patterns described [17, 22, 23, 26]. However, a Brazilian study found no patients with detectable ANCA in a cohort of 50 patients with confirmed TB [27]. However, taken together the studies have shown significant heterogeneity in results and methods employed, and conflicting results in regards to predominant ANCA patterns identified on IIF and/or confirmed on ELISA. Furthermore, these studies included varying proportions of patients on TB treatment, and later studies by Esquivel-Valerio et al. [25] and Elkayam et al. [24] subsequently identified that TB treatment itself appears to result in *de-novo* formation of ANCA antibodies when comparing data pre-TB treatment and post-TB treatment; therefore TB treatment may be a confounding factor in earlier studies.

In summary, pulmonary TB and GPA have many overlapping features clinically and radiologically which can pose a diagnostic dilemma. It is evident that treatment decisions need to be focused on the clinical presentation of patients and not ANCA testing alone. In patients with high-risk TB exposure history, TB should be actively excluded as an alternate diagnosis in patients with a positive ANCA before initiating high-dose immunosuppression. It appears that P-ANCA/MPO ANCA is most associated with pulmonary TB whilst, a combination of C-ANCA/PR-3 ANCA may be helpful in differentiating GPA from pulmonary TB. It is recommended that biopsies of lung lesions, sputum microscopy and multidisciplinary team input are sought as part of the workup when these two differentials are being considered.

## Data Availability

Data supporting our case presentation can be found in clinical documentation pertaining to patient’s clinical reviews by treating specialists (inpatient and outpatient clinical settings), imaging reports (sourced from IMPAX database), pathology results reported by Pathwest laboratories, Perth, WA. All data generated or analysed during this study are included in this published article [and its supplementary information files].

## Abbreviations

AAV: ANCA-associated vasculitides
ANCA: Anti-neutrophil cytoplasmic antibody
EGPA: Eosinophilic granulomatosis with polyangiitis
ELISA: Enzyme-linked immunosorbent assay
GPA: Granulomatous polyangiitis
IIF: Indirect immunofluorescence
MPA: Microscopic polyangiitis
MPO: Myeloperoxidase
PR3: Proteinase 3
